# Supplementary material for: A Comprehensive Multiomics Signature of Doxorubicin‐Induced Cellular Senescence in the Postmenopausal Human Ovary
Source: Aging Cell. 2025 Jun 1;24(8):e70111. doi: 10.1111/acel.70111 (PMC12341822; doi:10.1111/acel.70111)

Supplemental Figure 1: Optimization of IHC markers in native postmenopausal ovarian tissue

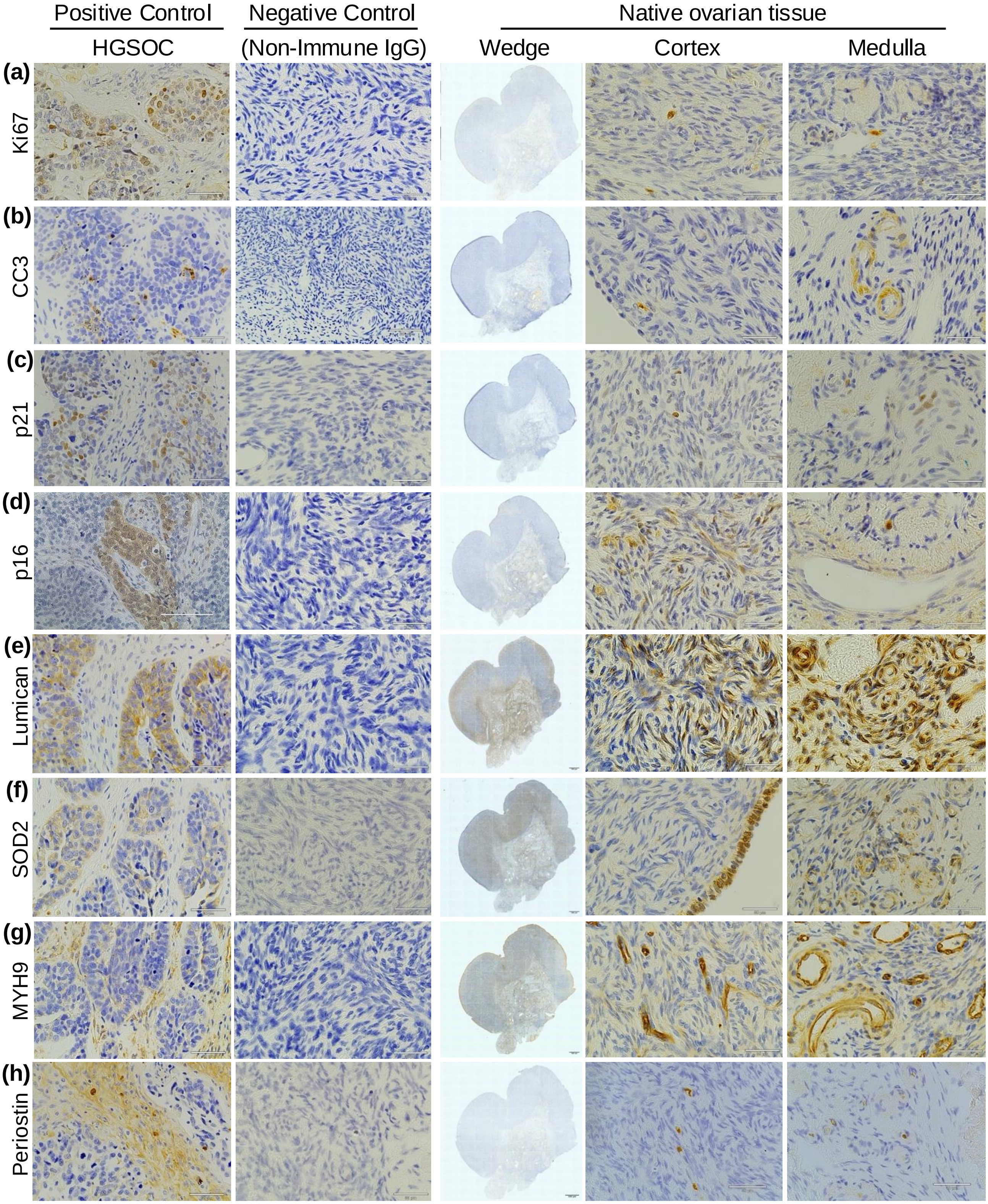

Supplemental Figure 2

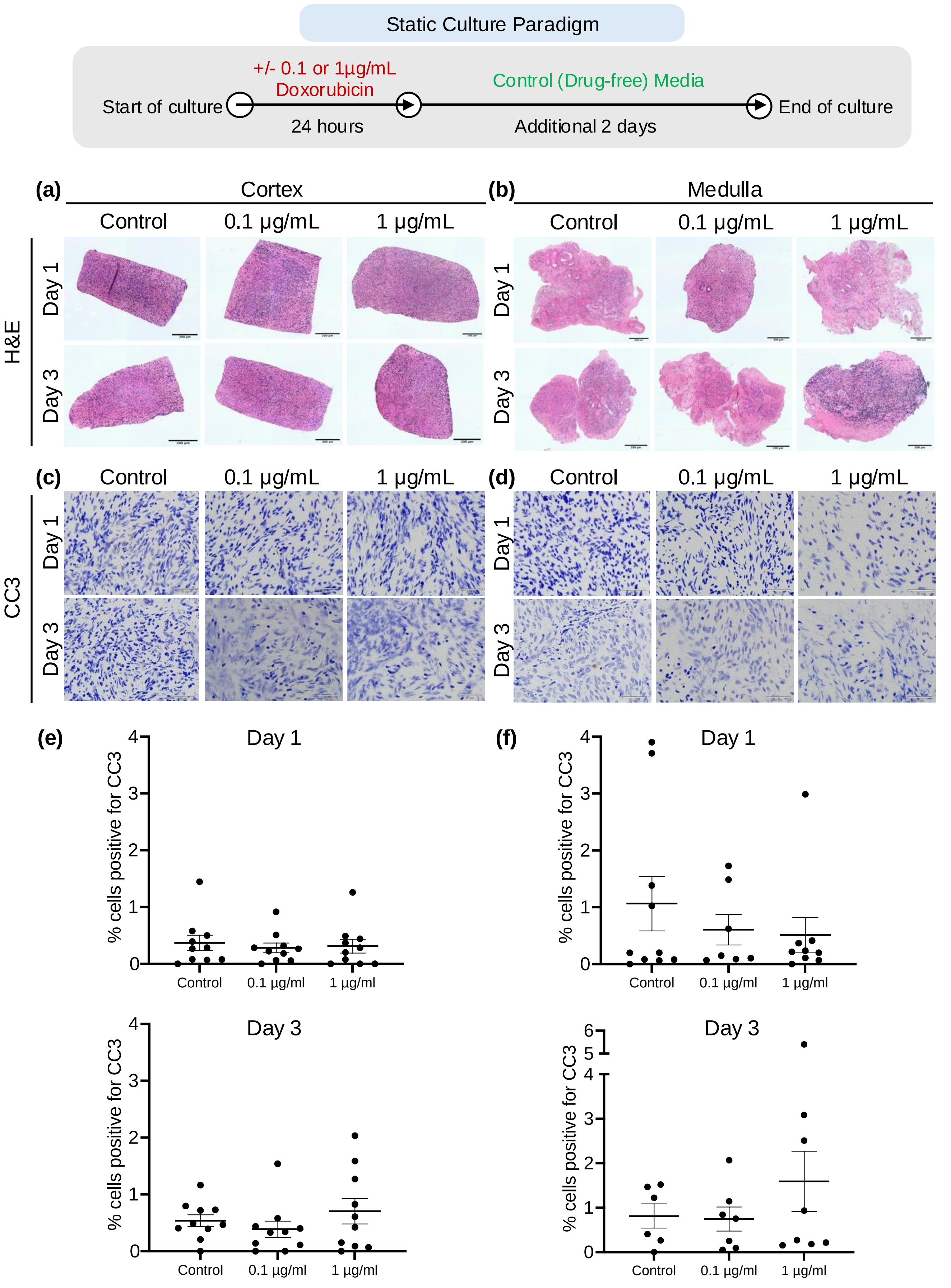

Supplemental Figure 3

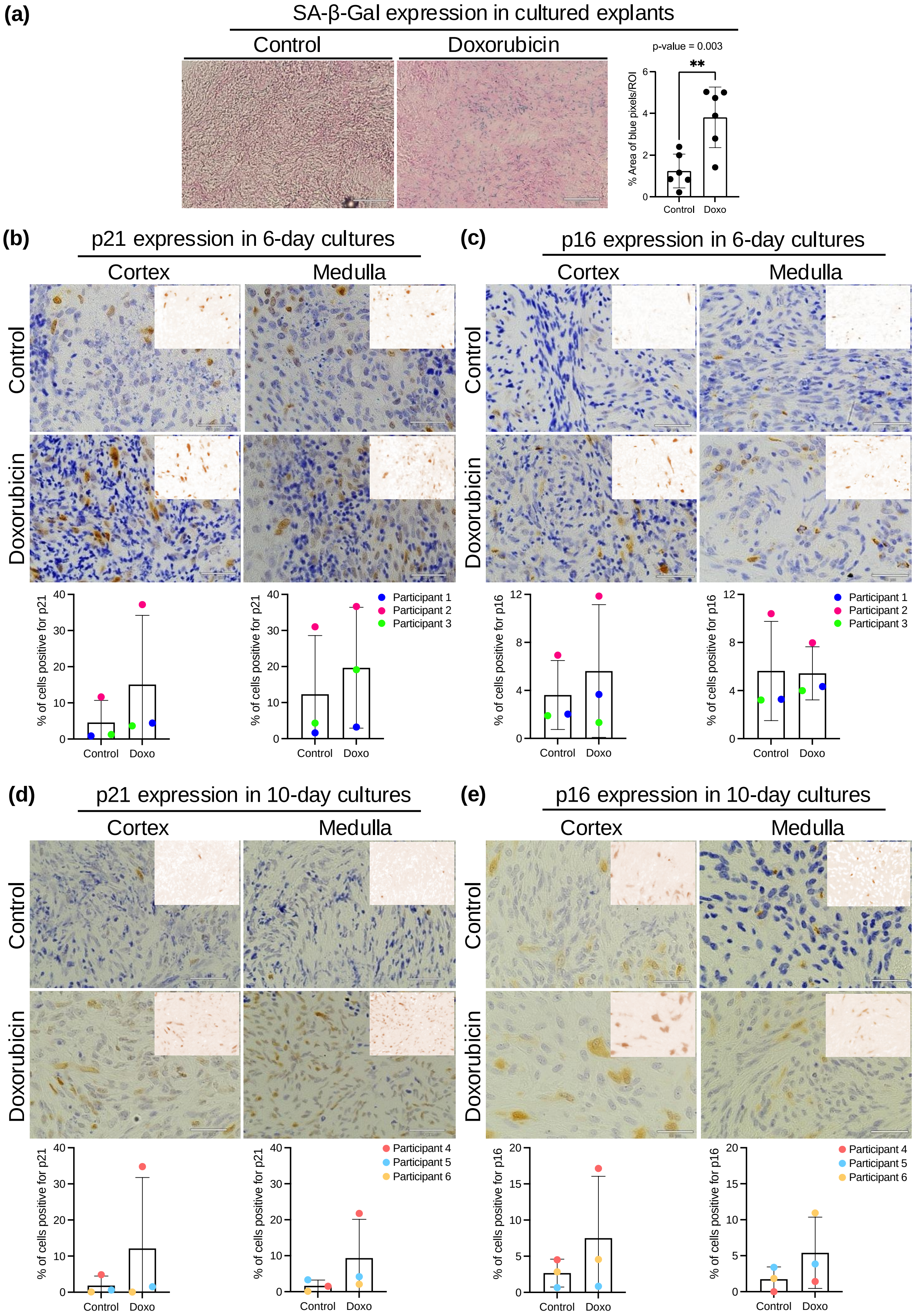

Supplemental Figure 4

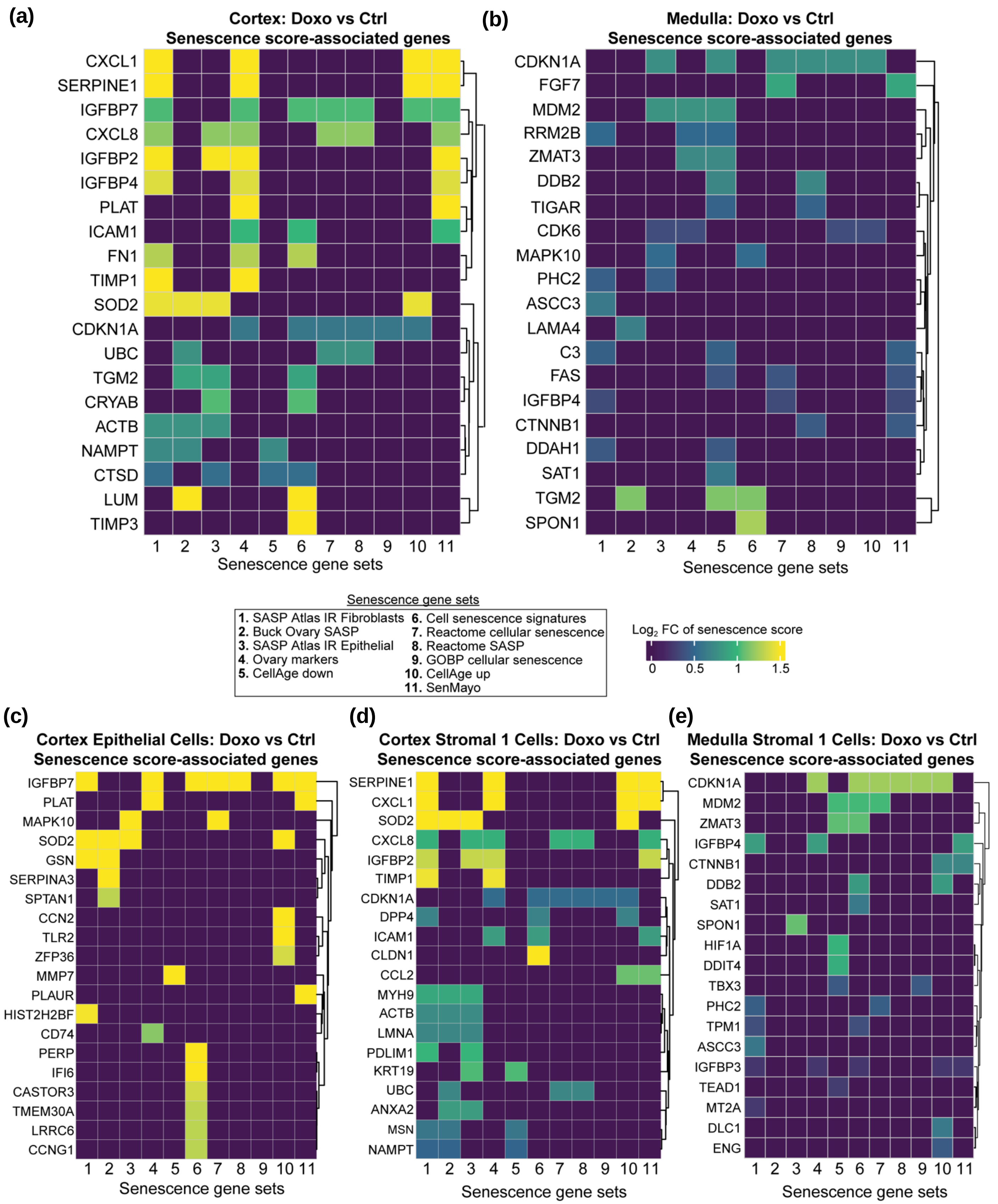

Supplemental Figure 5

(a)

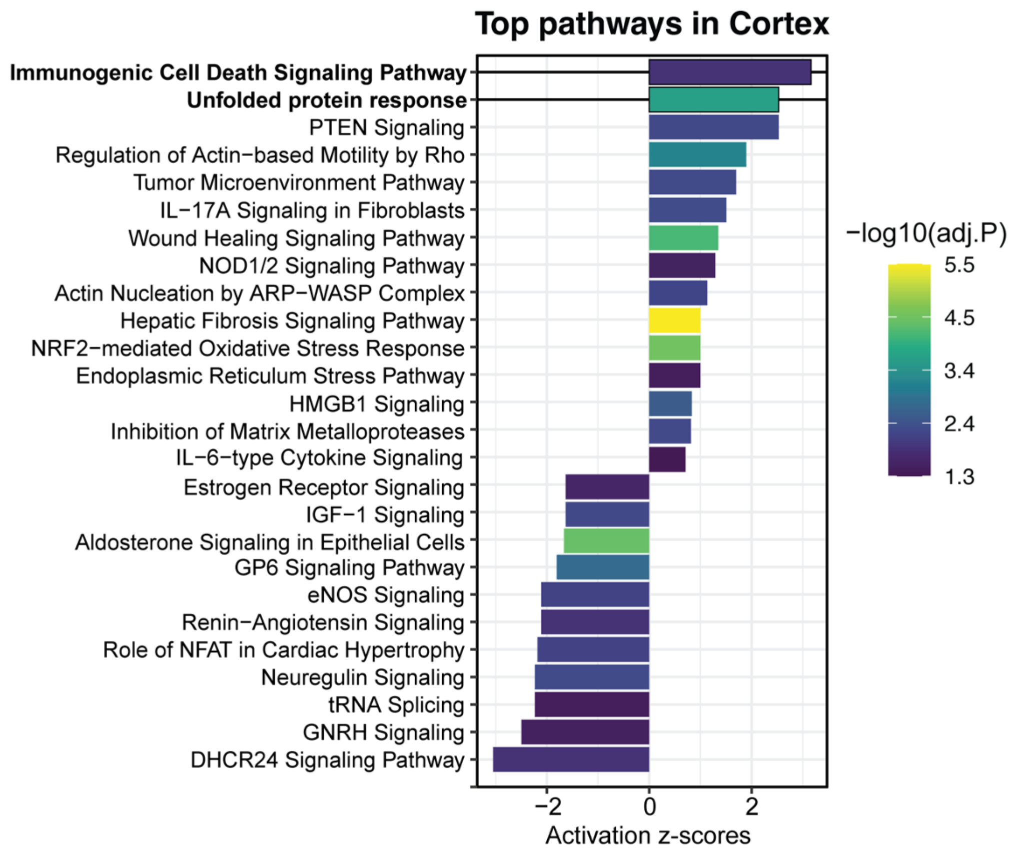

(b)

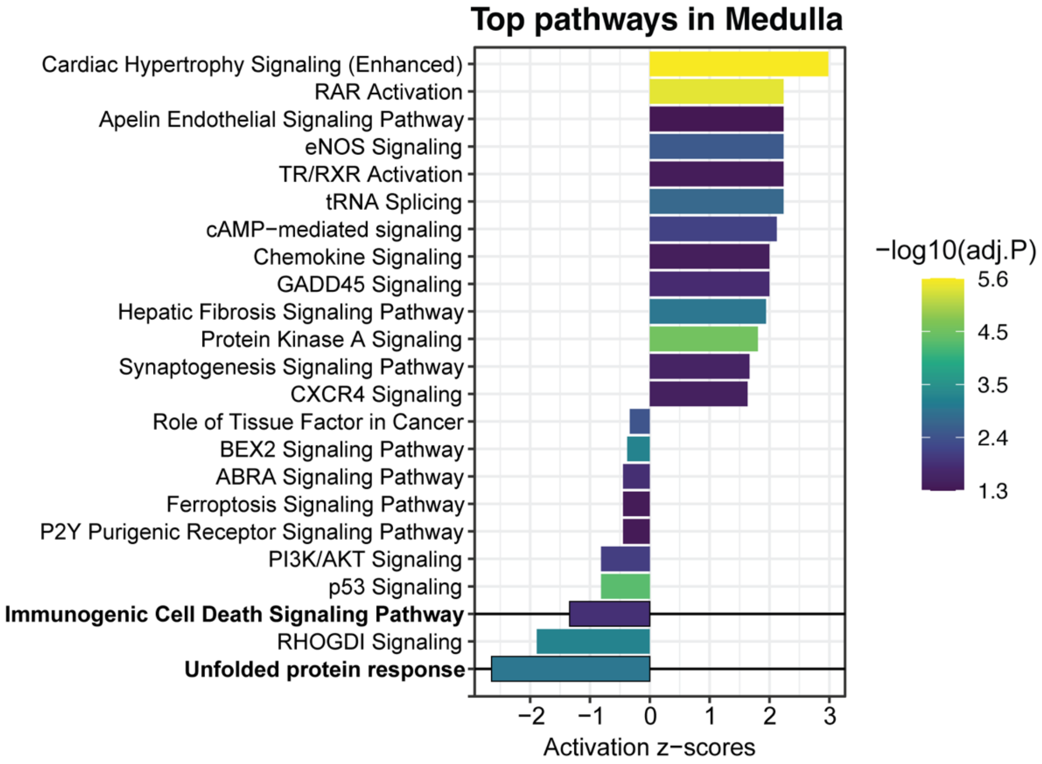

Supplemental Figure 6

(a) UMAPs showing Stroma1 and stroma 2 clusters across all 3 donors

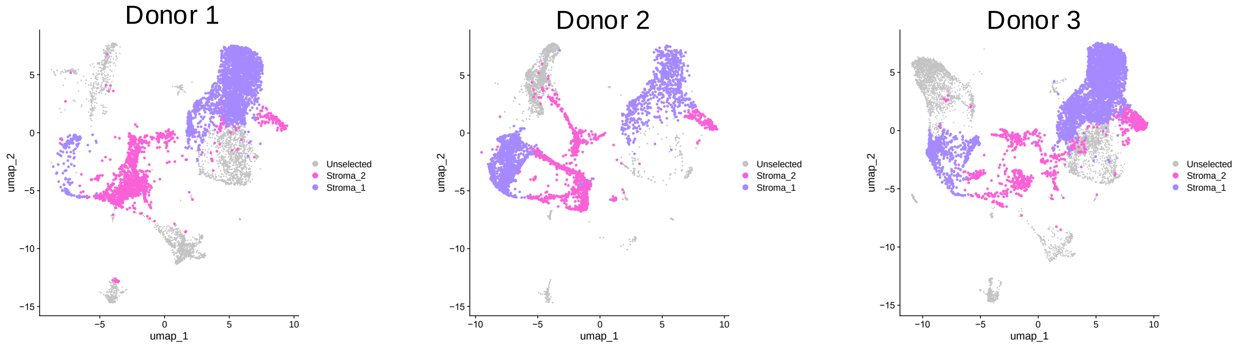

(b) Top pathways for Stroma 1 cluster

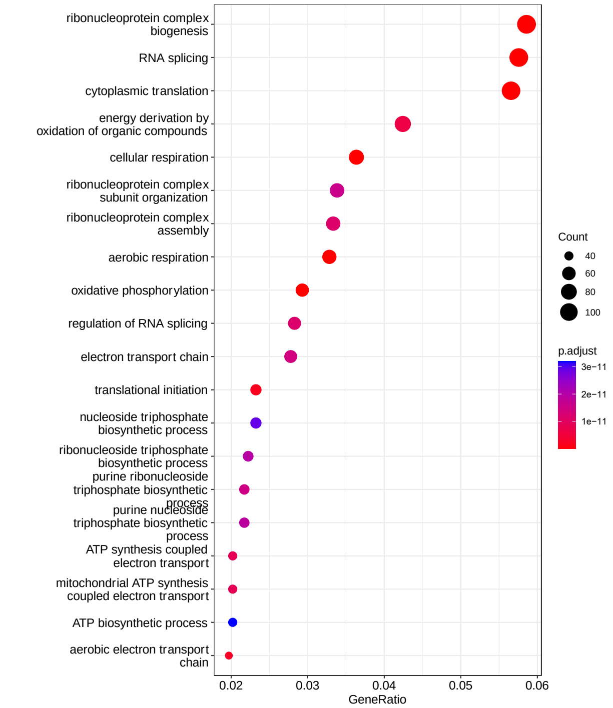

(c) Top pathways for Stroma 2 cluster

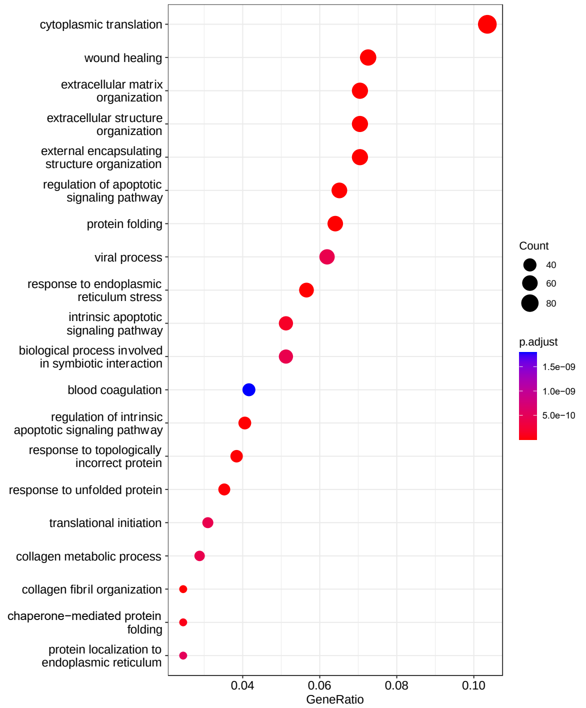

(d) Bar plot depicting frequency of cell types in control and doxo-treated explants

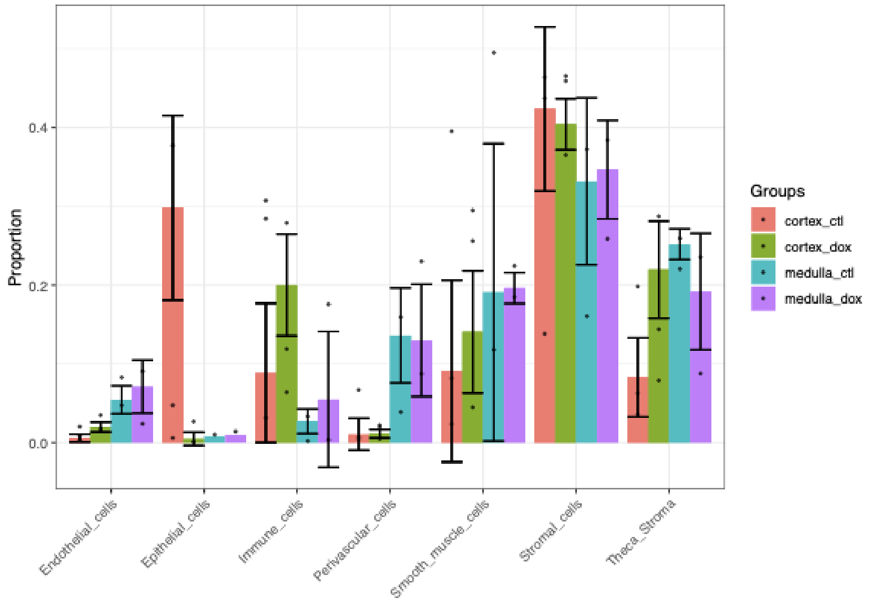

Supplemental Figure 7

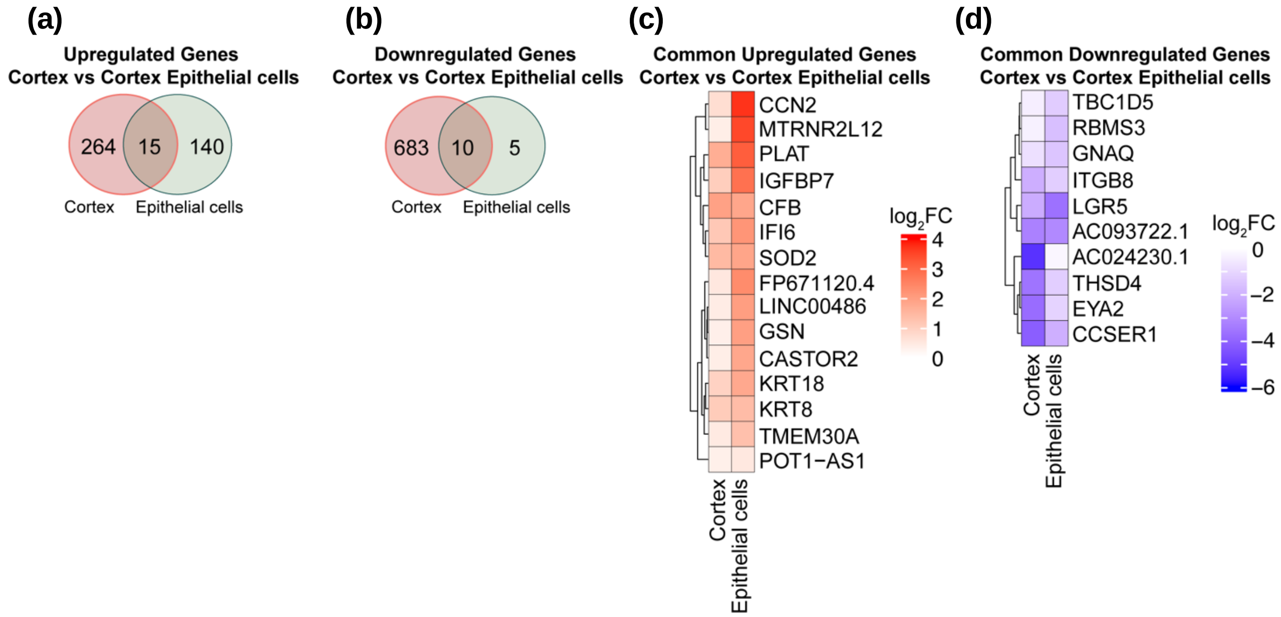

Supplemental Figure 8

(a)

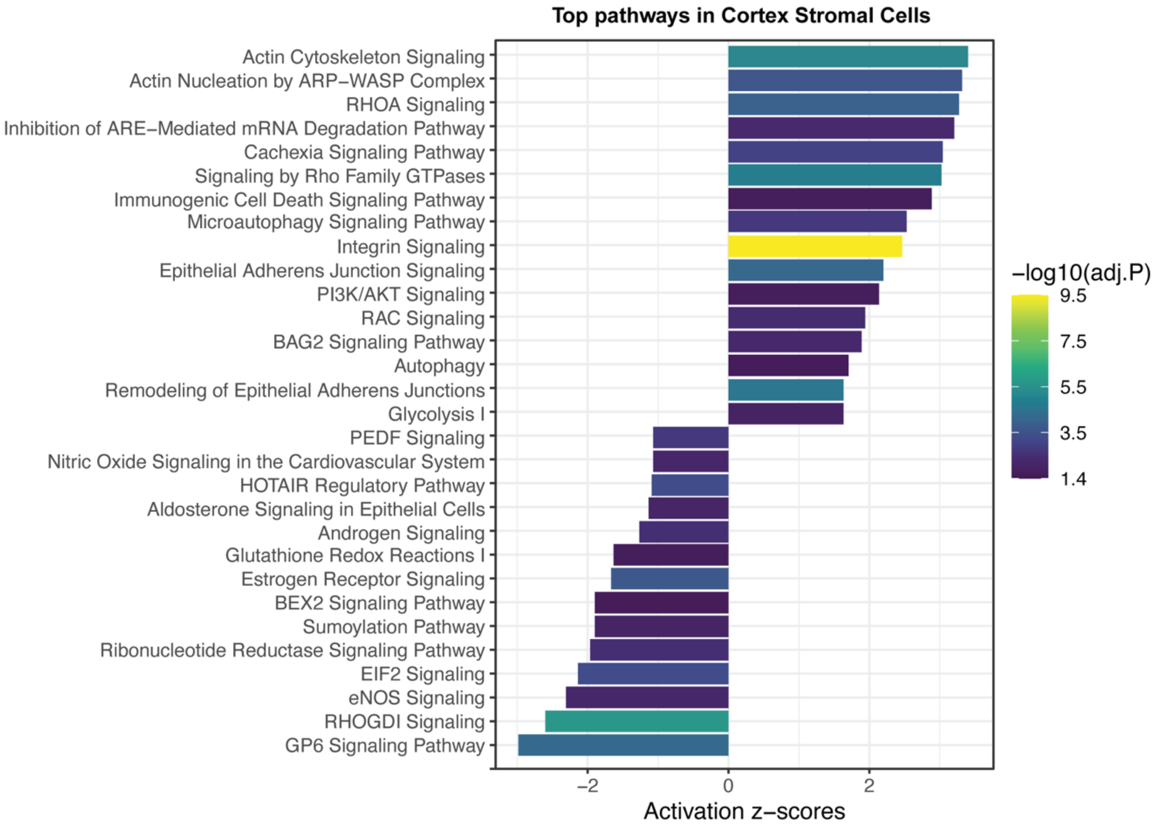

(b)

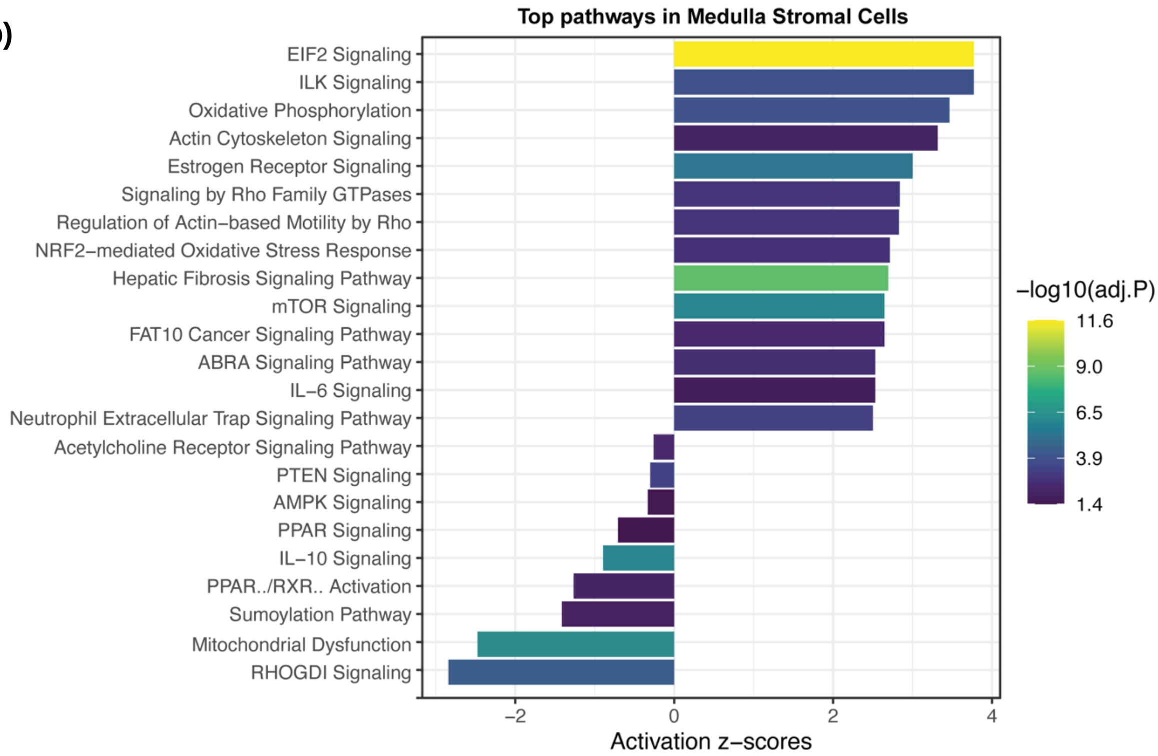

Supplemental Figure 9

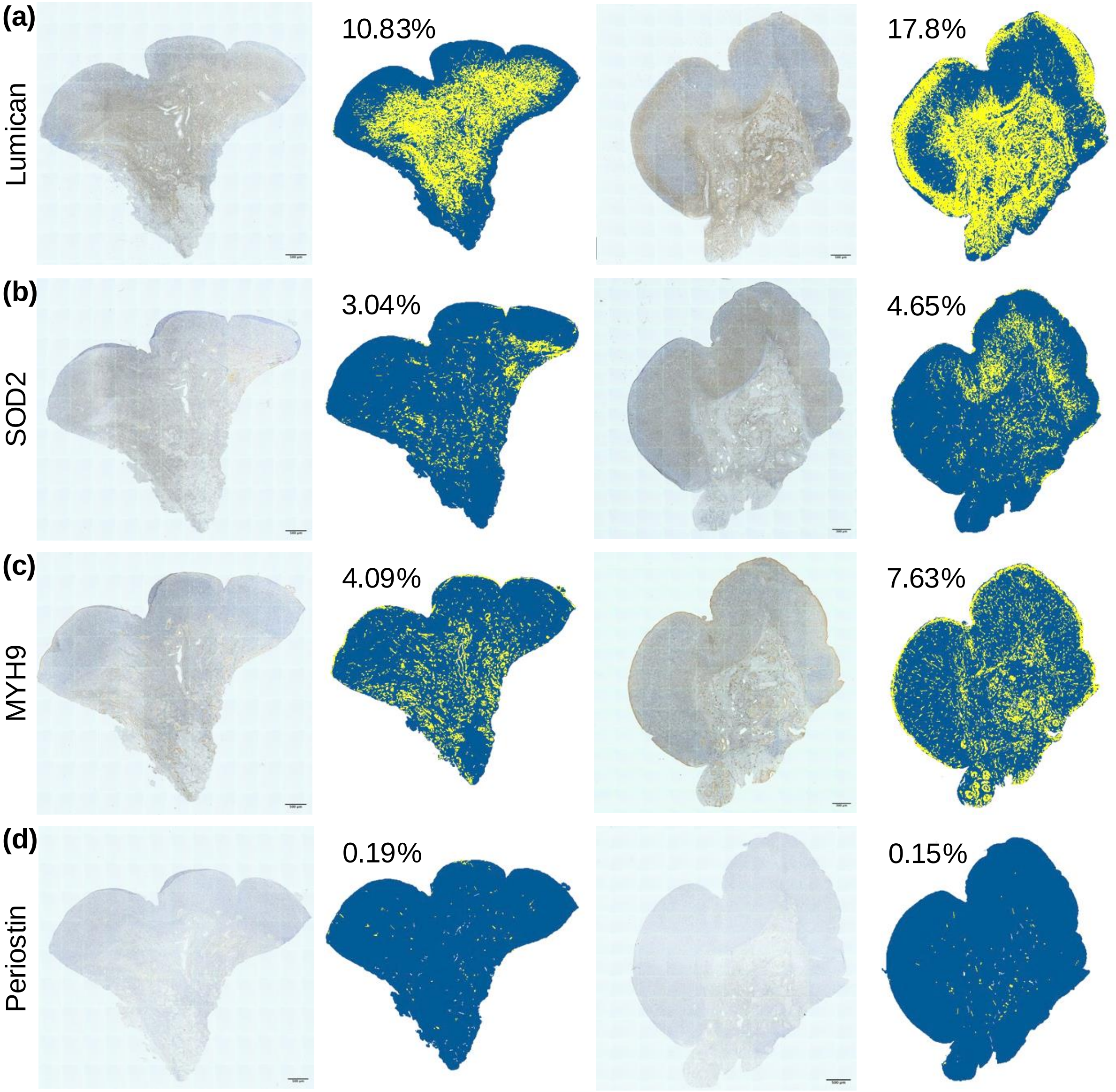

Supplement: Supplementary file 8 — Figure S1. Optimization of IHC markers in native postmenopausal ovarian tissue. All IHC markers were optimized in native postmenopausal ovarian tissue, along with relevant controls including High Grade Serous Ovarian Carcinoma (HGSOC; positive control) and negative controls (with nonimmune IgG). Ki67 (a), CC3 (b), and p21 (c) show very low to negative expression in the native postmenopausal ovarian tissue. p16 is expressed in discrete clusters in the cortex (d “Cortex”) and some expression in the medulla (d “Medulla”). Lumican (e) showed expression in both the cortex and medulla. SOD2 (f) showed a particularly strong expression in the ovarian surface epithelium (f “Cortex”). MYH9 (g) was expressed across both compartments particularly in vessel walls. Periostin (h) showed minimal expression in native tissue. Representative images from 73‐years‐old participant. Figure S2. Doxorubicin dose–response and explant viability in 3‐day cultures. H&E‐stained sections of human ovarian cortex (a) and medulla (b) explants cultured for 2 days after 24 h doxorubicin exposure (0, 0.1, 1 μg/mL). IHC for cleaved caspase‐3 (CC3) in human ovarian cortex (c, e) and medulla (d, f) explants on Day 1 (Day 1) and Day 3 (Day 3) of culture showed low levels of cellular apoptosis (p value > 0.05). Statistical significance was determined using an unpaired t‐test and ANOVA and p values < 0.05 were considered statistically significant (N = 4 participants, ages 57, 63, 68, and 70 years old). Scale bars correspond to 200 μm. Figure S3. Canonical markers of cellular senescence SA‐β‐Gal, p21CIP1, and p16INK4a trend toward increased expression with doxorubicin treatment. (a) SA‐β‐Gal staining in control versus doxorubicin‐treated cultured explants. Blue color is indicative of positive SA‐β‐Gal staining (p value‐ 0.003; N = 1 participant 69‐years‐old). p21CIP1 and p16INK4a showed a trend toward increased expression with doxorubicin exposure in 6‐day (2b,c; p value Cortex = 0.41 (p21) 0.61 (p16) and Med [file ACEL-24-e70111-s001.pdf]
